# Profile and course of early rheumatoid arthritis in Morocco: a two-year follow-up study

**DOI:** 10.1186/1471-2474-12-266

**Published:** 2011-11-23

**Authors:** Karima Benbouazza, Bahia Benchekroun, Hanan Rkain, Bouchra Amine, Fatiha Bzami, Leila Benbrahim, Ouafa Atouf, Malika Essakalli, Redouane Abouqal, Maxime Dougados, Najia Hajjaj-Hassouni

**Affiliations:** 1 Rheumatology department, Mohamed Vth University Souissi, El Ayachi hospital, Salé, Ibn Sina University hospital, Rabat, Morocco; 2 Blood transfusion and hemovigilance department, laboratory of immunology, Mohamed Vth University Souissi, Ibn Sina Universitary Hospital, Rabat, Morocco; 3 Laboratory of Biostatistics, Clinical and Epidemiological Research, Faculty of Medicine and Pharmacy, Mohamed Vth University Souissi, Rabat, Morocco; 4 Rheumatology B department, René Descartes University, UPRES-EA 4058, Cochin Hospital, Paris, France

## Abstract

**Background:**

This study aimed to establish the profile and the evolution of an early Rheumatoid arthritis **(RA) **cohort in the Moroccan population and also to search possible predictor factors of structural progression.

**Methods:**

Patients with early RA (< 12 months) were enrolled in a 2-year follow-up study. Clinical, biological, immunogenetic, and radiographical data were analyzed at study entry and at 24 months. Presence of radiographic progression was retained when the total score was superior to the smallest detectable difference (SDD) calculated to be 5.4 according the Sharp/van der Heijde (SVDH) method.

**Results:**

Fifty one patients (88.8% women, mean age of 46.9 [ 24-72 ] ± 10.8 years, mean disease duration of 24 [ 6-48 ] ± 13.9 weeks) were enrolled in this study. 68.6% were illiterate and 19.6% reported at least one comorbid condition. The mean delay in referral for specialist care was 140 [ 7-420 ] ± 43 days.

Thirteen patients (62.5%) were IgM or IgA RF positive. HLA-DRB1*01 and DRB1*04 alleles were present respectively in 11.8% and 45.1% of patients.

At baseline, 35.3% patients were taking corticosteroids and 7.8% were under conventional DMARDs.

At 24 months, 77.2% received a median dose of 5 mg/day of prednisone. Methotrexate (MTX) was the most frequently prescribed DMARD, being taken by 65.2% of patients. 13.6% of patients had stopped their DMARD because of socioeconomic difficulties.

Comparison of clinical and biologic parameters between baseline and 24 months thereafter revealed a significant global improvement of the disease status including morning stiffness, pain score, swollen joint count, DAS 28 and HAQ scores, ESR and CRP.

Sixteen patients (34.8%) were in remission at 2 years versus no patients at baseline; *P *< 0.001.

Forteen patients (27.5%) had at least one erosion at baseline. Radiographic progression occurred in 33.3% of patients and was associated in univariate analysis to swollen joint count (p = 0.03), total SVDH score (*P *= 0.04) and joint space narrowing score (*P *= 0.03). No independent factors of radiographic progression were shown by logistic regression.

**Conclusions:**

These study reports, provided for the first time in Morocco, a developing African country, a large amount of information concerning the profile and the course of early RA.

Patients who were receiving, for most of them, Methotrexate in monotherapy and low doses of corticosteroids, showed an improvement of all clinic and biologic disease parameters. Moreover, DAS remission was obtained in one third of patients and two thirds of the cohort had no radiographic progression at 2 years. No predictor factors of radiographic progression were found out.

These results should be confirmed or not by a large unbiased RA cohort which will give more relevant information about early RA characteristics and its course and will constitute a major keystone of its management.

## Background

Rheumatoid arthritis (RA) is the most common inflammatory chronic disease of the joints. It is characterized by disease activity and bone destruction resulting in joint destruction, functional impairment, and increased mortality [1-3]. In Morocco, RA has a major socioeconomic impact [4]. The low socioeconomic status of many patients and the inadequate social welfare and health insurance systems contribute to weight of this burden [4].

Early recognition of RA and intervention appear to be keys to optimal management of this chronic disabling disease [5]. There is a lack of research on early RA in Morocco. The purpose of this study is to establish the profile and the course of an early RA cohort between baseline and 2 years later. We would like also to determine in this prospective study if there are any possible predictor factors of structural progression.

## Methods

### Patients

A cohort of consecutive adults patients with RA aged between 18 and 70 years who suffered from the disease for less than 1 year was recruited in this study between January 2005 and December 2006. RA patients were diagnosed by rheumatologists with reference to the American College of Rheumatology (ACR) classification criteria [6]. Patients were followed by their rheumatologist and addressed for assessment as outpatients in El Ayachi hospital (public structure and referral hospital of Rheumatology in Morocco where hospitalizations and outpatient clinics are accessible for patients originary from different regions of our country) at baseline and 2 years thereafter. The choice of treatment in this cohort according to the protocol was left to the discretion of the treating physicians. All patients agreed to enrollment in a 2-year follow-up study and signed written informed consent. The study was approved by the ethics review.

### Patients' features and disease characteristics

Patients' features and disease characteristics were assessed at baseline and 2 years after. Thus, the following parameters were collected at study entry: age, sex, educational level, rural or urban provenance, adherence to a health insurance system, comorbidity (other chronic diseases requiring long-term medical care), disease duration, patient's assessment of pain (on a visual analog scale), number of swollen and tender joints (both by 28 joint count), extra-articular manifestations, functional status evaluated by the Moroccan adapted version of Health Assessment Questionnaire (HAQ) [7], erythrocyte sedimentation rate (ESR), C reactive protein (CRP) level, IgM or IgA rheumatoid factor (RF) positivity (20 IU/ml and 7 units/ml, respectively), Anti-citrullinated protein/peptide antibodies (ACPA) positivity using second generation anti-CCP assay (CCP2) of ELISA and HLA typing of class II (-DR and 15 DQ) tested by ''Polymerase chain reaction sequence specific primers'' (PCR-SSP).

We assessed patient disease activity at baseline and 2 years thereafter using the 28 joint disease activity score (DAS 28). Remission was defined by a DAS 28 score < 2.6 [8].

At 24 months, the clinical assessment included number of swollen and tender joints (both by 28 joint count) and also DAS 28, HAQ and pain scores. The laboratory investigations included ESR and CRP dosages.

Concerning treatments, we recorded at baseline and at 24 months doses of oral corticosteroids and type of DMARDs received by patients.

Radiographs of the hands and feet were obtained at baseline and 2 years thereafter. Structural damage was assessed by counting the number of erosions and grading the joint space narrowing (JSN) according to the Sharp and van der Heijde method [9].

All radiographs were scored separately by two experienced assessors, who were unaware of the clinical data of the patients. The *x *ray pictures were read in their chronological sequence. Each observer performed every measurement twice. Intra-observer reliability was evaluated by linear regression between the outputs of the first and second measurements, and intra-observer error was calculated as the absolute difference between measurements [10]. Radiographic inter-observer agreement was (ICC) = 0.89 and intra-observer ICC varied between 0.93 and 0.92. Those findings indicated good intra-observer and inter-observer reliability and are in line with literature [11].

The smallest detectable difference (SDD), defined as that number greater than the measurement error of progression, was determined using the metric 95% limits of agreement method of Bland and Altman [10,12]. Using this method, SDD was calculated to be 5.4. Thus, presence of radiographic progression was retained when the total score was superior to the SDD leading to a simple division into radiological progression and no progression.

### Statistics

Descriptive statistics of the patients and disease characteristics were calculated. Student's t test and the chi-squared test were used as appropriate to compare the evolution of clinic, biologic and radiographic parameters between outset and 24 months thereafter. Outcome variables were dichotomized into qualitative variables: presence or absence of radiologic progression, presence or absence of remission.

Univariate analysis tested most of the clinical, biologic, immunologic and genetic factors that were previously reported to be possibly related to structural progression in RA.

Multivariate logistic regression analyses were also conducted. A statistical significance level of *P *< 0.05 was used in all statistical tests performed. Analyses were performed using the SPSS program (version 13.0; SPSS Inc., Chicago, IL, USA).

## Results

### Patients' and disease' features at baseline

Fifty one patients (45 women, 6 men) were enrolled in this study. Six patients (1 patient died, 5 refused further follow-up) were lost to follow-up at 2 years. Data on 45 patients were thus available for analysis 2 years after their inclusion in the study.

Of all participants, 68.6% were illiterate and only 3.9% had been to high school. Five patients (9.8%) adhered to a health insurance system. Overall, 10 cases (19.6%) reported at least one comorbid condition (arterial hypertension (5.8%) hypercholesterolemia (3.9%), diabetes mellittus (5.8%) and hypothyroïditis (3.9%)).

At baseline, the mean age of patients and the mean disease duration were respectively 46.9 [24-72] ± 10.8 years and 24 [6-48] ± 13.9 weeks. The mean delay in referral for specialist care was 140 [7-420] ± 43 days. Thirteen patients (62.5%) were IgM or IgA RF positive. Positivity of ACPA was noted in 56.6% of patients.

HLA-DRB1*01 and DRB1*04 alleles were present respectively in 11.8% and 45.1% of patients. At baseline, DAS 28 and HAQ scores were respectively 6.9 [3.7- 9.9] ± 1.3 and 2.2 [0.7-3] ± 0.6.

At study entry, eighteen patients (35.3%) were already taking corticosteroids and 4 patients (7.8%) were under conventional DMARDs (Methotrexate (n = 1) and Methotrexate associated to Chloroquine (n = 3)).

At 24 months assessment, thirty four patients (77.2%) received a low dose of prednisone with a median dosage of 5 mg/day. During the study, all patients were receiving conventional DMARDs in monotherapy. Methotrexate (MTX) was the most frequently prescribed DMARD, being taken at a dose of 15 mg once a week by 65.2% of patients and followed by Sulfasalazine and Chloroquine (10.8% for each of them). At the 24 months assessment, 6 patients (13.6%) had stopped the DMARD for socioeconomic difficulties. None of patients received combination therapy or biologic agents.

Table 1 shows the patients' and disease' characteristics at baseline.

**Table 1:** Patient and disease characteristics at baseline

Baseline variable	Value
**Sex, % women**	88.2
**Age, years**	46.9 [24-72] ± 10.8
**Provenance, % urban/rural**	74.5/25.5
**Education level**	
**Analphabet, %**	68.6
**High level school, %**	3.9
**Comorbidity, %**	18.6
**Disease duration, weeks**	24 [6-48] ± 13.9
**Referral to specialist delay, days**	140 [7-420] ± 100
**Stiffness duration, mn**	138 [15-360] ± 76
**Extraarticular manifestations, %**	1.8
**ESR, mm/hour**	57.2 [5-115] ± 25.8
**CRP, mg/liter**	26.8 [0-96] ± 22
**RF positivity, %**	62.5
**Antibody positivity, %**	7.8
**ACPA positivity, %**	56.6
HLA-DRB1*04, %	45.1
HLA-DRB1*01, %	11.8
**Patient global assessment (0-10)**	3.4 [0-8] ± 1.6
**Physician global assessment (0-10)**	6.2 [1-10] ± 1.7
**Patient pain assessment (0-10)**	6.6 [1-10] ± 1.8
**Tender joint count (0-28)**	20.4 [8-48] ± 10.1
**Swollen joint count (0-28)**	9.6 ± 6.5
**HAQ (0-3)**	2.2 [0.7-3] ± 0.6
**Presence at least of one erosion, %**	27.5
**Total SVDH score**	1 (0-5)*
**Erosion score**	0 (0-1)*
**Joint space narrowing score**	1 (0-3)*
**Treatment received**	
**Corticosteroids, %**	35.3
**DMARD, %**	7.8

Values are presented as mean or percentage.

* Values are presented as median and quartiles.

RA: Rheumatoid Arthritis: RF: Rheumatoid Factor; HAQ: Health Assessment Questionnaire; DAS 28: Disease Activity Score; ESR: erythrocyte sedimentation rate; CRP: C-reactive protein; DMARDs: Disease-Modifying Anti-Rheumatic Drugs.

### Early RA evolution between baseline and at 24 months

Table 2 shows a comparison of clinical and biologic parameters of the RA between inclusion and 24 months later. These results revealed a significant global improvement of the disease status including morning stiffness, pain score, swollen joint count, DAS 28 and HAQ scores, ESR and CRP.

**Table 2:** Comparison of clinical and biologic parameters of the early RA between baseline and 2 years

Parameters	Baseline	2 years of follow-up	*P*
**Morning stiffness**	120 (60-180)*	10(0-75)*	< 0,001
**Patient pain assessment**	70 (50-80) *	20(0-50)*	< 0,001
**Swollen joint count**	9 (4-14) *	0(0-2)*	< 0,001
**DAS 28**	6,9 [3.7-9.9] ± 1.3	3,7 [0.5-9.2] ± 1.9	< 0,001
**HAQ Score**	2,2 (1.6-2.7) *	0,3(0-1,4)*	< 0,001
**ESR**	57,2 [5-115] ± 25,8	28,6 [2-85] ± 23,5	< 0,001
**CRP**	24 (12-48) *	11,1 (2-12)*	< 0,001

Values are presented as mean or percentage.

* Values are presented as median and quartiles.

### Disease activity and remission

In RA patients, the mean DAS 28 decreased significantly from an initial mean score of 6.9 [3.7-9.9] ± 1.3 in range of high disease activity to 3.7 [0.5- 9.2] ± 1.9 into the range of moderate disease activity; *P *< 0.001. Sixty patients (34.8%) were in remission versus no patients at baseline; *P *< 0.001.

### Structural damage and radiological progression

Forteen patients (27.5%) had at least one erosion at baseline. At 2 years, the median of total SVDH, erosions, and joint space narrowing scores increased respectively from 1(0-5) to 4(1-10); *P *< 0.001, from 0(0-1) to 0(0-2); *P *< 0.001, and from 1(0-3) to 3(1-8) ); *P *< 0.001; (Table 3).

**Table 3:** Comparison of structural damage between baseline and 2 years

Scores	Median and inter quartiles Baseline N = 51	Median et inter quartiles 24 months N = 45	p
**Erosions**	0(0-1)	0(0-2)	**< 0,001**
**Joint space narrowing**	1(0-3)	3(1-8)	**< 0,001**
**Total SVDH**	1(0-5)	4(1-10)	**< 0,001**

N = patients' number

In this inception cohort of 45 patients with early RA, 33.3% showed progressive joint damage over 2 years (their total score was superior to the SDD).

Table 4 shows a comparison of outset parameters between the two groups according to the presence of radiological progression at the 2 year- follow-up. In this univariate analysis, radiological progression risk was significantly associated with swollen joint count (*P*= 0.03), total SVDH score (*P *= 0.04) and joint space narrowing score (*P *= 0.03). No association was noted for any other variables. Logistic regression showed that there were no independent factors of radiographic progression.

**Table 4:** Determinant factors of structural progression by univariate analysis

Items at baseline	Radiologic Progression		
	OR	IC 95%	*P*
**Age**	0.9	(0.92-1.03)	0.4
**Sex**	0.7	(0.10-4.86)	0.7
**Delay in referral to specialist**	1	(0.99-1.00)	0.7
**Morning stiffness**	1	(0.9-1)	0.1
**Swollen joint count (0-28)**	1.1	(1-1.3)	**0.03**
**DAS 28**	1.4	(0.8-2.5)	0.2
**HAQ**	2.6	(0.8-8.5)	0.1
**RF positivity**	1.2	(0.3-4.4)	0.8
**ESR**	1	(0.9-1)	0.1
**CRP**	1	(0.9-1)	0.3
**ACPA**	1.7	(0.4-6.3)	0.4
HLA-DRB1*04	1	(0.5-5.7)	0.4
HLA-DRB1*01	2.2	(0.4-12.3)	0.4
**Total SVDH score**	1.3	(1-1.7)	**0.03**
**Joint space narrowing score**	1.4	(1.1-1.9)	**0.04**
**Erosions score**	1.8	(0.7-2.7)	0.4

Comparing clinical and biologic characteristics of progressor and non-progressor patients at the 24 months, showed no significant (NS) differences in swollen joint (2.8 vs 1.1; *P*: NS), tender joints (11.5 vs 6.5; *P*: NS), ESR (31.2 vs 27.8; *P*: NS), HAQ (0.7 vs 0.6; *P*: NS), DAS 28(3.9 vs 3.6; *P*: NS), and patient pain assessment (3.1 vs 2.3; *P*: NS).

## Discussion

This prospective study constitutes a real-life design and provides, for the first time in Morocco and to our knowledge in Africa, the profile of early RA in a developing African country. It shows also that there are no predictor factors of radiographic progression after 2 years of follow-up.

The age onset of RA in Morocco has been reported in a previous retrospective study to be lower than in western countries [13]. In the same way, recent data on the profile of patients with RA in 8 clinics in North Africa (Morocco and Egypt) in the QUEST-RA (Quantitative Standard Monitoring of Patients with RA) database, shows that patients in North Africa are younger than patients in other countries [14]. Then, our findings of similarity of with RA age onset in western countries should be taken with caution and may not be extended to the Moroccan population.

The optimal approach to early RA management requires early referral of patients with arthritis to specialists and early diagnosis of the disease using mainly clinical and serological investigations [15]. In our study population, referral to specialists was delayed since it took nearly 20 weeks. Therefore, more awareness of the generalists is required to promote early referral to a specialist in order to guarantee the patient's early diagnosis and therapeutic intervention. This early diagnosis challenge remains greater for developing countries where RA management is still not considered as a public health priority even if it constitutes an enormous socioeconomic burden [4,16].

ACPA has acquired for many years a great interest in early RA diagnosis. It's to be noted that our ACPA percentage result was similar to that reported by Meyer and al in their cohort [17]. Regarding the genetic concern, association with Human Leukocyte Antigen (HLA)-DRB alleles, implicated in the aetiopathogenesis of Rheumatoid Arthritis (RA), is found to be different in various ethnic groups [18]. In the Moroccan population, there is only one study which focused on evaluating the distribution of class I and class II HLA genes. In this controlled data, Atouf et al suggest that DRB1*04 predisposes to RA in the Moroccan population [19].

At the 2 year assessment, 6 patients (13.6%) have stopped the DMARD due to socioeconomic difficulties. Our finding highlights once more another frequent problem in developing countries inherent to socioeconomic difficulties encountered in chronic diseases management [16]. Rkain et al reported that 62% of Moroccan patients with established RA reported bad observance to treatments because of financial difficulties [4]. In the same way, an earlier data on therapeutic maintenance of MTX in RA reported that 14% of Moroccan patients stopped MTX for socioeconomic reasons [20].

The significant improvement of the disease status including clinical and biologic parameters is in concordance with the literature findings [21,22].

Practically a third of patients were in remission after this period whereas there were no patients at baseline. Remission rates range from 3% to 68% [23] in RA studies depending on selection of remission criteria, patient selection, duration of the follow-up period, and therapies.

Our study has provided large information on structural progression. Forty patients (27.5%) had at least one erosion at baseline. Previous studies have widely demonstrated that joint damage in RA occurs early in the disease process [24]. Patients with duration of symptoms less than 3 months may have already evidence of destruction [25]. In the ESPOIR cohort, 20% of 813 patients with mean disease duration of 107 days had hand or foot erosions [26].

Two thirds of our patients did not show any significant radiographic progression after 2 years of follow-up.

Comparison of this result with literature is difficult because of the differences in the study design, patients and disease features.

Both nonprogressor and progressor patients showed significant improvement of clinical and biologic baseline parameters. Those findings are in line with previous data showing that radiological progression occurs despite a clinically and biologically acceptable disease control [27,28].

Traditional therapy with a single DMARD is often insufficient to prevent a poor long-term outcome of rheumatoid arthritis (RA) [29]. Trials in RA patients have shown that treatment with combination therapy and/or biologic agents is more effective in preventing radiographic joint damage than MTX as monotheray [29-31]. Our progressor patients would probably benefit strongly from DMARD combinations or biologic agents.

Many predictor factors of structural progression were reported in literature [32-35]. According to most of the published reports, long-term progression of joint damage was best predicted at baseline by the presence of IgM or IgA rheumatoid factor, high ESR or CRP level, number of swollen joints, presence of anti-CCP antibodies, HLA-DRB1*0401 and 0404 genes, HAQ and early radiographic erosions. In our study, univariate analysis has initially shown that the radiographic progression was significantly associated with three baseline factors: swollen joint count, total SVDH and Joint space narrowing scores. However, none of those factors have been retained as being independent factors of radiologic progression after multiple logistic regression. This observed lack of association between radiographic progression and different factors shown in literature could be due to a lack of power.

Many limitations should be recognized in our study. First, we recognize that choosing the ACR 1987 classification criteria for RA to recruit patients may be criticized and could be a bias recruitment, possibly misjudging the baseline structural damage. But at our study onset, a few years ago, Early arthritis was "ill-defined": in some studies a disease duration of up to three years was accepted, whereas other protocols required a much shorter maximum duration of symptoms [36]. In addition, because of technical and financial considerations, we have not included in the design study regular assessment throughout the 2 years of follow-up. Thus, we were unable to calculate the score of radiographic progression. Finally, we should perhaps have included in database more details on socioeconomic conditions to analyze their possible contribution to the course of the disease in our context.

More than a research subject, this study gave us as clinicians the opportunity to find out the early RA profile in the Moroccan context.

## Conclusion

This study reports, for the first time in Morocco, a developing African country, a large amount of information concerning the profile and the course of early RA.

Patients, receiving, for most of them, Methotrexate in monotherapy and low doses of corticosteroids, showed an improvement of all clinical and biological disease parameters. Moreover, remission was obtained in one third of patients and two thirds of the cohort had no radiographic progression at 2 years. No predictor factors of radiographic progression were found out probably in relation with the small number of patients.

The findings of this study also highlight the difficulties of early RA in developing countries. The delay in referral to specialists and interruption of DMARD due to financial difficulties are the most important problems encountered in our context.

The small size of our sample and the absence of regular monitoring of clinical, biological and radiographical parameters during the course of the disease are the most important limitations of our study.

The best way to investigate the course of early RA in the Moroccan population would be through a prospective longitudinal study, following an unbiased cohort of patients with early RA with regular clinical, biological and radiographical assessments for many years. Such multicentric inception cohort of early arthritis with a larger sample will give more precious informations about early RA characteristics and course and will constitute a major keystone in the management of this disabling disease in our country. Such a prospective study is already ongoing in Morocco.

## Competing interests

The authors declare that they have no competing interests.

## Authors' contributions

KB, NHH and MD conceived the study and supervised its design and execution. FB, BB, AB and LB were involved in sample collection. HR, RA, BB and LB and did data management and statistical analyses. OA and ME carried out HLA typing. HR wrote the paper with input from all investigators. All authors were involved in drafting manuscript and revising it for critically important content. All authors have read and approved the final manuscript.

## Pre-publication history

The pre-publication history for this paper can be accessed here:

http://www.biomedcentral.com/1471-2474/12/266/prepub
